# Flap Optimization Using VISUMAX 800 Femtosecond Laser for Laser-Assisted In Situ Keratomileusis

**DOI:** 10.3390/jcm15186980

**Published:** 2026-09-09

**Authors:** Abdullah Virk, Sanjana Molleti, Hanna Pawlowski, Randle M. Uyeda, Jason D. H. Chung, Phillip C. Hoopes, Majid Moshirfar

**Affiliations:** 1 College of Medicine—Phoenix, University of Arizona, Phoenix, AZ 85004, USA; 2 Hoopes Moshirfar Research Center, Draper, UT 84020, USA; 3 Department of Ophthalmology and Visual Neurosciences, University of Minnesota, Minneapolis, MN 55455, USA; 4 Department of Ophthalmology, Loma Linda School of Medicine, Loma Linda, CA 92350, USA; 5 John A. Moran Eye Center, University of Utah, Salt Lake City, UT 84112, USA; 6 Utah Lions Eye Bank, Murray, UT 84107, USA

**Keywords:** LASIK, Visumax 800, OBL, opaque bubble layer, refractive surgery, flap optimization, cone size

## Abstract

**Background:** Opaque bubble layers (OBLs) are a common intraoperative complication during flap creation in femtosecond laser-assisted LASIK (FS-LASIK). This study aims to evaluate how cone and flap sizes can be optimized to minimize OBL risk in VISUMAX 800 FS-LASIK. **Methods:** This was a retrospective chart review of patients who underwent LASIK with the VISUMAX 800. Eyes were grouped by cone size and laser settings into S cone/M settings and M cone/L settings, in addition to OBL status (OBL vs. non-OBL). Generalized estimating equations were used to account for inter-eye correlation. Flap–cone edge gap was quantified from intraoperative images using FIJI (version 2.17.0, ImageJ). **Results:** OBL developed in 17 of 226 eyes (7.5%). The majority of OBLs (15/17) occurred in the M cone with L setting group. Eyes that developed OBLs had larger WTWs (*p* < 0.001) and larger mean flap diameters (*p* < 0.001). Among eyes with M cones and L settings, image analysis revealed that the average gap between flap and cone edge was significantly larger in the OBL group (*p* = 0.007). **Conclusions:** Our findings support the use of VISUMAX 800 in FS-LASIK and suggest that OBL formation may be associated with the distance between flap and cone edges, with larger average flap–cone gaps observed in eyes that developed OBL.

## 1. Introduction

Since the advent of femtosecond lasers, the diversity in devices available to physicians has steadily increased, with each femtosecond laser device on the market differing in procedural speed, corneal flap thickness, accuracy, predictability, efficacy, and complication rates [1]. A notable complication unique to femtosecond laser-assisted LASIK (FS-LASIK) is the development of an opaque bubble layer (OBL), which is formed from the trapped residual gas bubble in the corneal stroma after the collapse of cavitation bubbles formed from plasma expansion [2]. Newer femtosecond laser devices such as the VISUMAX 800 (Carl Zeiss Meditec, Jena, Germany) utilize lower pulse energy and have shown reduced rates of OBL formation [3]. Therefore, selection of a femtosecond laser device should not only consider operative efficiency but also clinical outcomes and risk of intra- and postoperative complications.

The VISUMAX 500 femtosecond laser is commonly used for LASIK and small incision lenticule extraction (SMILE) after being FDA-approved in September 2016, and has been associated with excellent safety, predictability, and efficacy profiles [4,5]. However, there have been reports of significant OBL rates with LASIK ranging from 5 to 72.59% and SMILE ranging from 0.72 to 51.8% depending on flap dimensions and surgical technique. Additional limitations include longer suction times and complications such as increased risk of transient light sensitivity syndrome [6,7,8,9,10,11,12]. These limitations prompted the development of VISUMAX 800, a second-generation high-speed femtosecond laser currently FDA-approved for SMILE [13]. Compared to its predecessor, the VISUMAX 800 femtosecond laser has faster laser, docking, and suction times (13 s vs. 34 s) [8,14]. Nevertheless, existing literature on the performance and complications of this device in LASIK is sparse. This study aims to identify methods to optimize flap creation with respect to cone size in order to minimize the risk of OBL occurrence in VISUMAX 800 FS-LASIK.

## 2. Methods

This study was a retrospective chart review of patients who underwent LASIK with the VISUMAX 800 femtosecond laser at a refractive surgery center between June 2025 and June 2026. This study was approved by the Hoopes Vision Ethics Committee and adhered to the tenets of the Declaration of Helsinki. The Biomedical Research Alliance of New York (BRANY) Institutional Review Board (#A20-12-547-823) approved this study. Written informed consent was obtained from all patients.

Included patients were over 18 years of age and were treated for myopia with or without astigmatism. Patients were excluded if they had previously undergone refractive surgery or if they had had ocular disease, hyperopia, or clinically significant dry eyes. Preoperative data collected included demographic characteristics, uncorrected distance visual acuity (UDVA) and corrected distance visual acuity (CDVA), and manifest refraction. Keratometry and central corneal thickness (CCT) measurements were obtained from Pentacam (Oculus, Wetzlar, Germany) corneal tomography. White-to-White (WTW) corneal diameter was calculated as the average of NIDEK (Gamagori, Japan) and Galilei G4 (Ziemer, Port, Switzerland) measurements when both were available. Intraoperative data, including complications (OBL), laser time, suction time, flap thickness, and flap diameter, were also collected. OBL was defined as any opacification within the flap diameter, with severity increasing proportionally to surface area covered. Postoperative follow-up examinations were performed at 1 day, 1 week, 1 month, and 3 months after surgery, with UDVA and CDVA collected at all visits. Additionally, postoperative refraction was collected at both 1 and 3 months.

Refractive outcomes were reported using 9 standard graphs to assess safety, efficacy, predictability, stability, and astigmatic outcomes. Astigmatic vector analysis was conducted through the AstigMATIC software using the Alpins method [15].

### 2.1. Surgical Method

All procedures were completed by a single surgeon (MM). The VISUMAX 800 femtosecond laser (Carl Zeiss Meditec AG, Jena, Germany) was used for LASIK flap creation. Initial surgical settings were configured to SMILE Pro and subsequently aborted manually to enable LASIK treatment. Flap creation using the VISUMAX 800 was performed at 2 MHz in a spiral pattern. Flap thickness ranged from 100 to 110 µm, while flap diameters ranged from 8.0 to 8.8 mm. The following laser parameters were used: flap cut spot distance of 3.5 μm, flap cut track distance of 3.5 μm, side cut spot distance of 2 μm, side cut track distance of 2 μm, 90° side-cut angle, laser energy of 175 nJ (energy index: 35) for both side and flap cut, hinge position of 90 degrees, hinge width of 3.62 mm, and hinge angle of 50°. Flap diameters and cones were determined based on WTW. In this study, cone modification techniques were utilized to increase flap diameter for each cone size, with M cone settings being used in S cones (flap diameters ≤ 8.4 mm) and L cone settings being used in M cones (flap diameters ≥ 8.5 mm).

Corneal ablation was performed using the Allegretto EX500 (Alcon, Erlangen, Germany) excimer laser. The most recent nomogram for the system, Datagraph-med version 5.80 (Datagraph, Chapel Hill, NC, USA), was utilized with an optical zone of 6.50 mm and a 9.00 mm transition zone.

Postoperative management consisted of one drop of moxifloxacin 0.5% ophthalmic solution in both eyes following the procedure. Patients continued moxifloxacin 0.5% and prednisolone acetate 1% combo eye drops every two hours for 1 day and then four times per day for one week. Preservative-free artificial tears were recommended as needed.

### 2.2. Statistical Analysis

Statistical analyses were performed in Microsoft Excel (V16.106; Microsoft Corp., Redmond, WA, USA), Python (V3.14.3; Python Software Foundation), and R (V4.5.3; R Foundation for Statistical Computing, Vienna, Austria). Generalized estimating equations (GEEs) with robust standard errors were utilized to account for inter-eye correlation among patients contributing bilateral eyes. An exchangeable working correlation structure was used for postoperative visual and refractive outcomes. For variables that were near-identical between fellow eyes (WTW, flap diameter, keratometry, and pachymetry), an independence working correlation structure was utilized to avoid unstable estimation of inter-eye correlation approaching 1. A *p*-value of less than 0.05 was considered statistically significant.

### 2.3. Image Analysis

**Figure 1:**
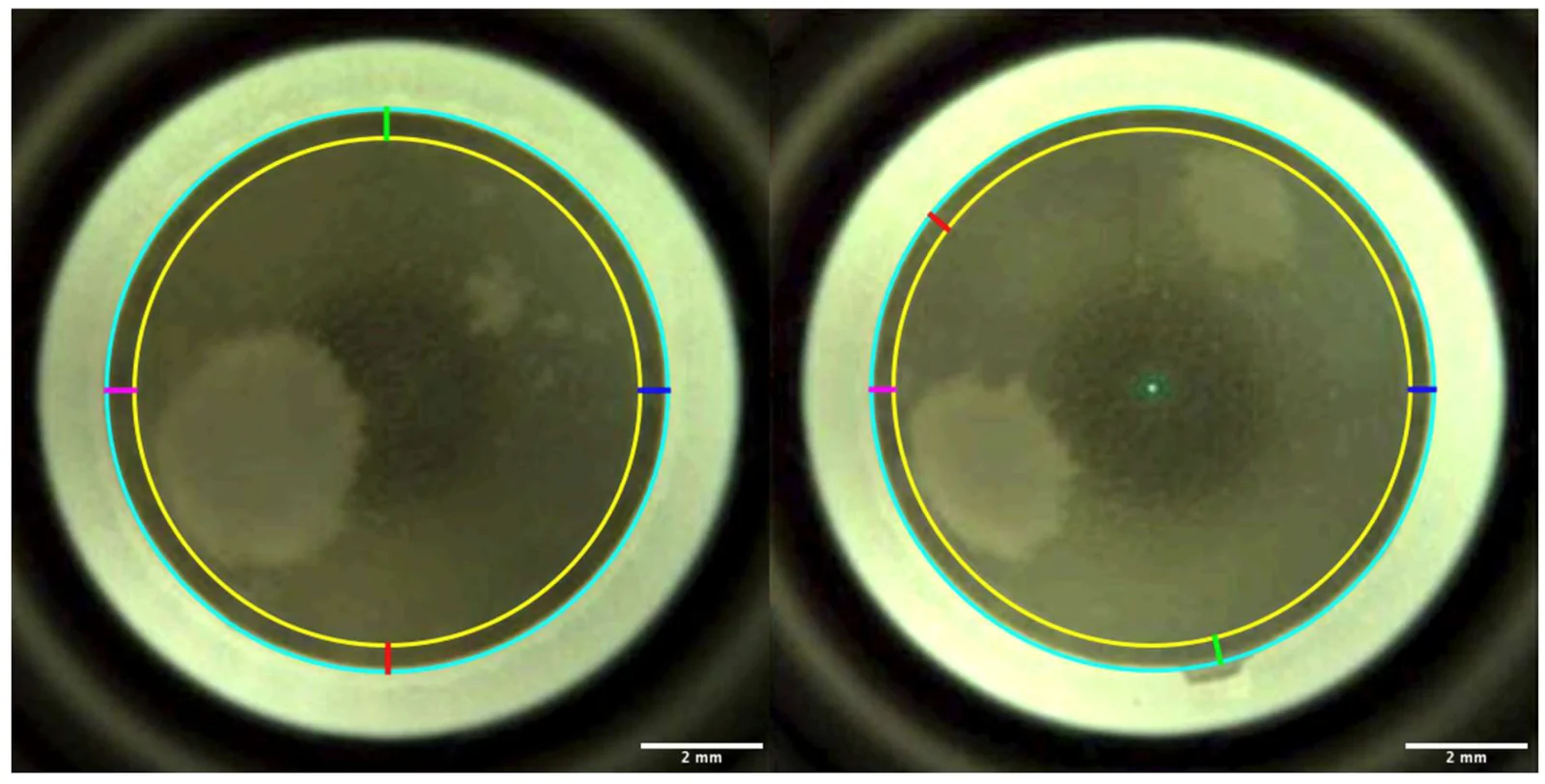
Fiji Image analysis of flap–cone edge gap. The yellow ellipse reflects the flap diameter while the cyan ellipse represents the cone diameter. The temporal gap, nasal gap, longest radial gap, and shortest radial gap are represented by the magenta line, blue line, green line, and red line, respectively.

Cone sizes were measured manually by three researchers (AV, JC, and MM) independently using calipers, who compared their measurements and reached consensus. The S cone measured 8.7 mm, and the M cone measured 9.3 mm. Image analysis was conducted with Fiji (version 2.17.0; ImageJ, Madison, WI, USA), an open-source distribution of the ImageJ software [16]. The image set was divided and reviewed by two researchers (AV and JC). Flap pictures were de-identified and imported into Fiji, and then a reference scale was set using the known measured cone diameter. Distances were initially measured in image pixels and converted to millimeters using this scale. For each image, ellipses were manually fitted to overlap the visible cone and flap boundaries (Figure 1). Images with uncertainty regarding flap or cone boundary identification were jointly reviewed to reach consensus, with an additional investigator (MM) available for cases that needed a tiebreaker. The average flap–cone gap was then calculated as the arithmetic mean of the radial flap–cone distances circumferentially. Additionally, radial asymmetry analysis utilized exact nasal and temporal gaps (blue and magenta lines, respectively; Figure 1), which were single distances between the nasal/temporal flap and cone edge. To allow for a more representative measurement for each region, a sector average gap of 30° for nasal and temporal sides was also calculated.

## 3. Results

A total of 226 eyes from 117 patients were included in this study, of whom 109 contributed bilateral eyes. Mean age was 34 ± 8.5 years (range 20–58 years), and 55% were female (Table 1). Mean preoperative sphere and cylinder were −2.81 ± 1.81 D (range −7.75–0.75 D) and −1.08 ± 1.02 D (range −5.00–0.00 D), respectively. Mean WTW was 11.93 ± 0.38 mm (range 11.00–12.90 mm), mean flap thickness was 100 ± 1.2 µm (range 100.00–110.00 µm), and mean flap diameter was 8.27 ± 0.19 mm (range 8.00–8.80 mm). At later timepoints, the number of eyes gradually decreased to n = 137 at 1 month and n = 70 at 3 months, due to loss to follow-up, follow-up completed at external locations, or surgery performed shortly before the end of the data collection period.

### 3.1. Safety and Efficacy

**Figure 2:**
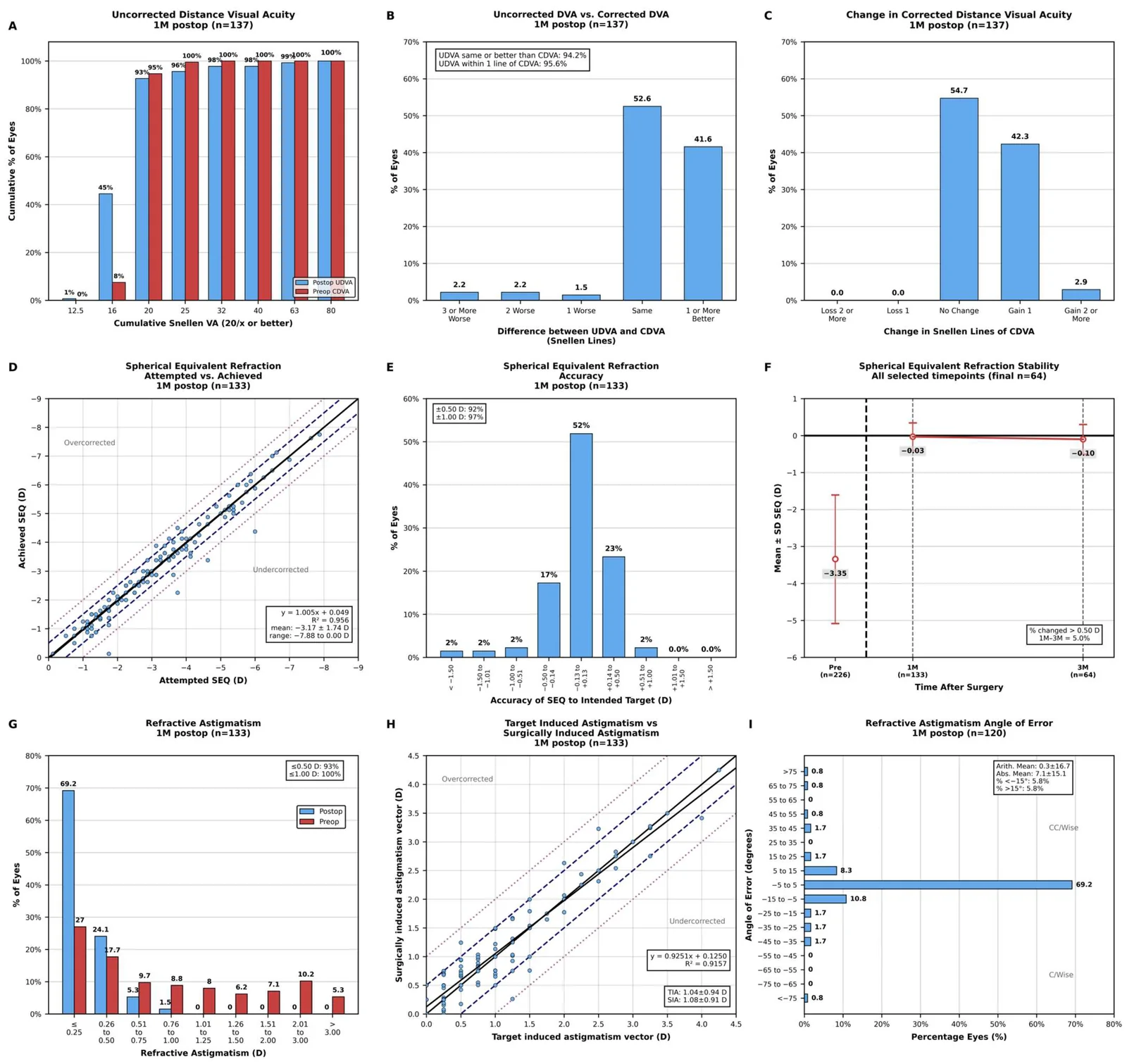
Standard 9 graphs for 1 month postoperative refractive outcomes after VISUMAX 800-assisted LASIK, with (**A**) uncorrected distance visual acuity (UDVA) outcomes, (**B**) postoperative UDVA versus preoperative CDVA, (**C**) change between preoperative and postoperative CDVA, (**D**) attempted versus achieved spherical equivalent, (**E**) accuracy of spherical equivalent, (**F**) spherical equivalent stability, (**G**) residual astigmatism, (**H**) target induced astigmatism versus surgically induced astigmatism, and (**I**) angle of error.

**Figure 3:**
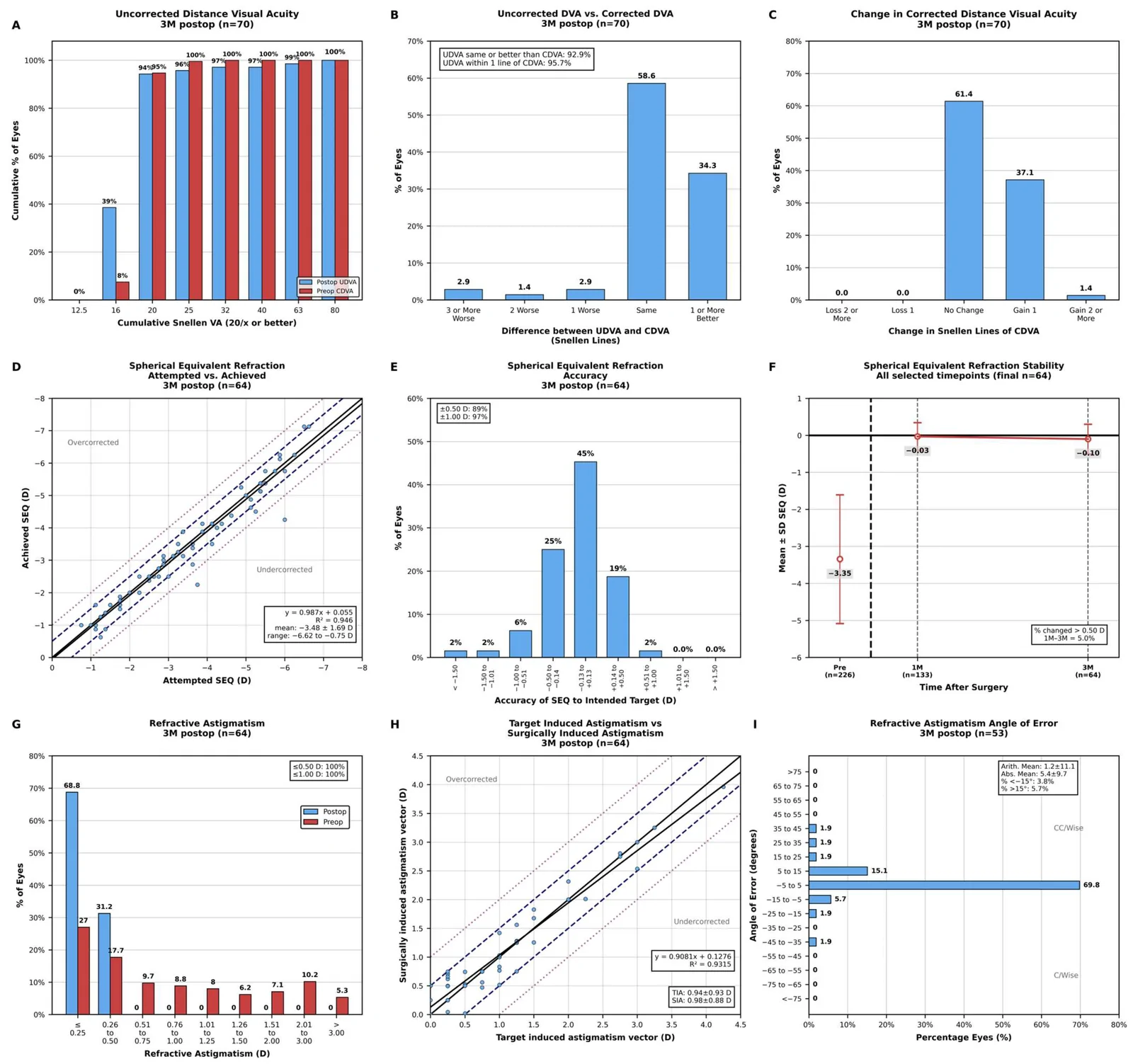
Standard 9 graphs for 3 month postoperative refractive outcomes after VISUMAX 800-assisted LASIK, with (**A**) uncorrected distance visual acuity (UDVA) outcomes, (**B**) postoperative UDVA versus preoperative CDVA, (**C**) change between preoperative and postoperative CDVA, (**D**) attempted versus achieved spherical equivalent, (**E**) accuracy of spherical equivalent, (**F**) spherical equivalent stability, (**G**) residual astigmatism, (**H**) target induced astigmatism versus surgically induced astigmatism, and (**I**) angle of error.

At 1 and 3 months postoperatively, 93% and 94% of eyes had a UDVA of 20/20 or better (Figure 2A and Figure 3A). The postoperative UDVA was the same as or better than preoperative CDVA in 94.2% at 1 month and 92.9% of eyes at 3 months (Figure 2B and Figure 3B). All patients had no change or gain of 1 or more lines of CDVA at both timepoints (Figure 2C and Figure 3C). The safety index was 1.14 ± 0.17 at 1 month and 1.13 ± 0.17 at 3 months, while the efficacy index was 1.10 ± 0.22 and 1.08 ± 0.21, respectively.

### 3.2. Predictability and Stability

The scatterplots (Figure 2D and Figure 3D) depict attempted versus achieved SEQ, demonstrating strong correlation (R^2^ = 0.96 at 1 month and 0.95 at 3 months). At 1 month postoperatively, 92% of eyes were within ± 0.5 D and 97% within 1 D of the intended target. At 3 months postoperatively, 89% of eyes were within ± 0.5 D and 97% within ± 1.0 D (Figure 2E and Figure 3E). Moreover, between 1 and 3 months, refractive stability was high, with only 5% of eyes demonstrating a change over 0.5 D (Figure 3F).

### 3.3. Astigmatism Analysis

**Figure 4:**
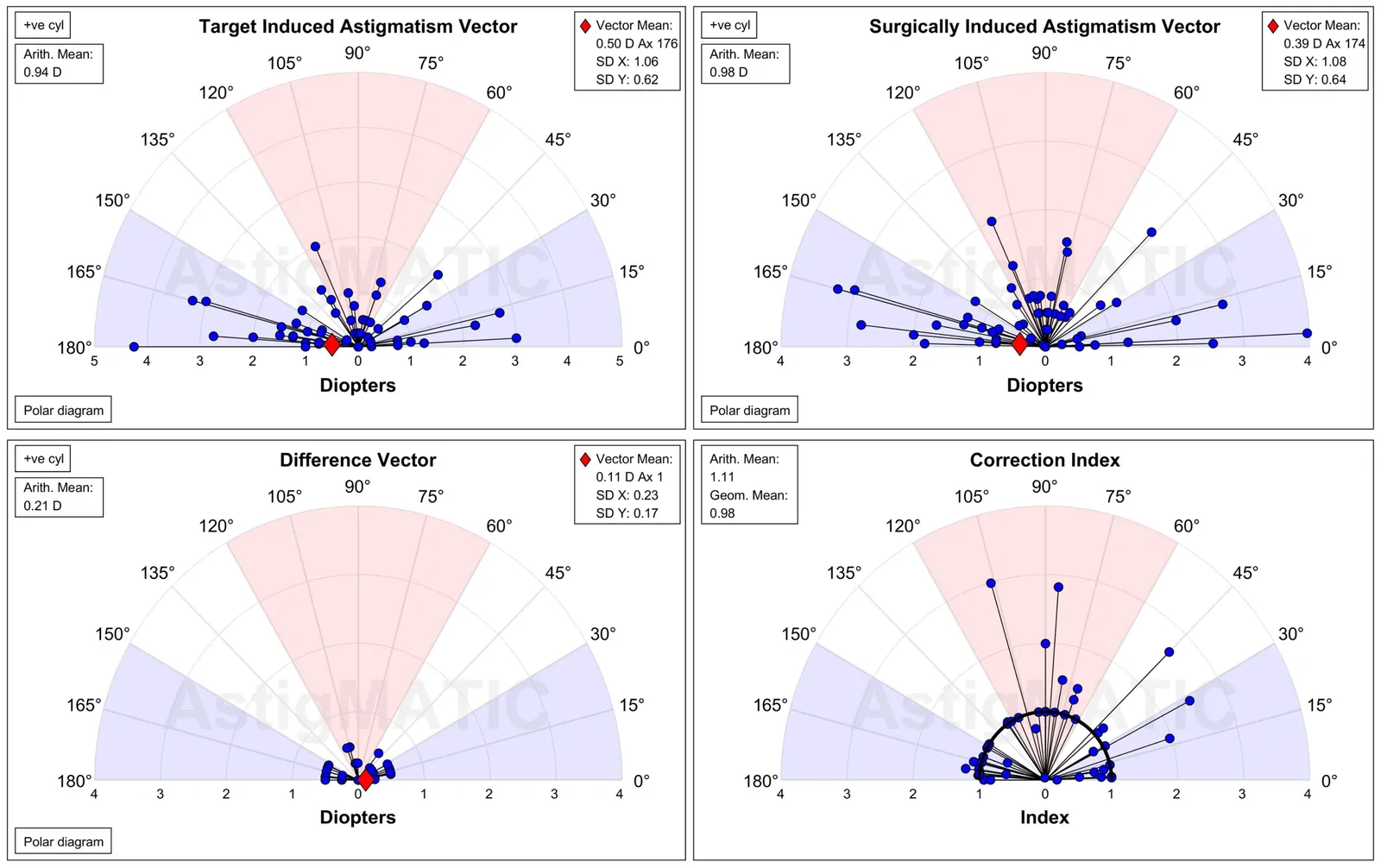
AstigMATIC plots of refractive astigmatism 3 months postoperatively with target induced astigmatism vector, surgically induced astigmatism vector, difference vector, and the correction index.

A total of 93% of eyes 1 month postoperatively achieved a refractive astigmatism of ≤0.5 D, with 100% of eyes 3 months postoperatively having a refractive astigmatism of ≤0.5 D (Figure 2G and Figure 3G). There was also a strong relationship between target-induced astigmatism and surgically induced astigmatism for both 1 and 3 months (R^2^ = 0.92 and 0.93, respectively; Figure 2H and Figure 3H). At 3 months postop, vector analysis shows that the mean correction index was 1.11, indicating slight overcorrection (Figure 4).

### 3.4. Complications

**Figure 5:**
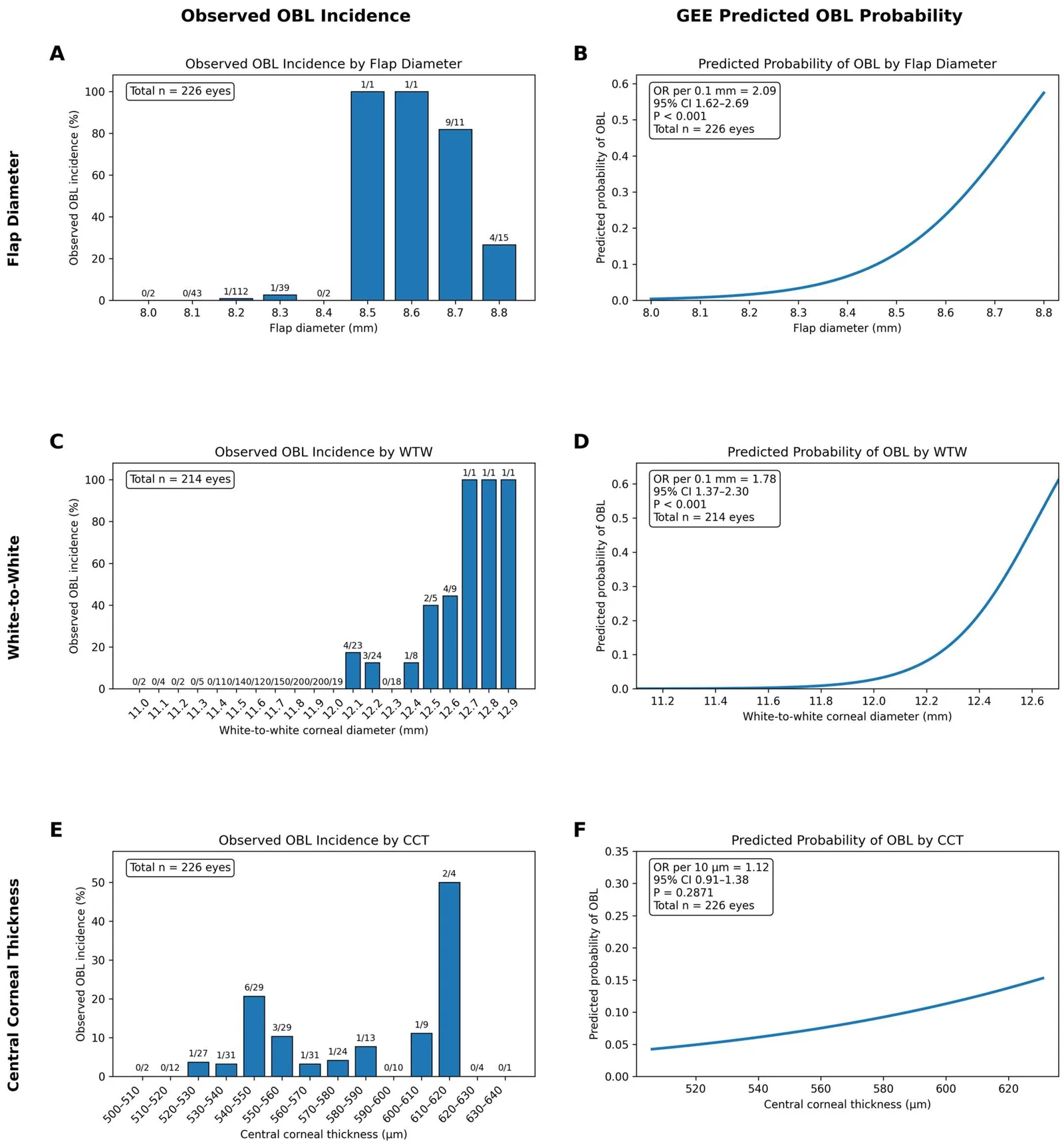
OBL incidence and unadjusted predicted probabilities by corneal flap diameter (**A**,**B**), WTW (**C**,**D**), and CCT (**E**,**F**).

The most common complication was the formation of opaque bubble layers (OBL) in the flap area (17/226; 7.5%), which were more common in larger flap diameters and WTW values (Figure 5A,C). In a GEE logistic regression model, the unadjusted predicted probability of OBL formation increased significantly with larger flap diameters and WTW measurements (*p* < 0.001; Figure 5B,D). In a complementary GEE comparison of OBL versus non-OBL groups, the OBL group had larger mean WTW measurements (12.42 ± 0.26 mm vs. 11.89 ± 0.36 mm; *p* < 0.001) and larger mean flap diameters (8.65 ± 0.17 mm vs. 8.23 ± 0.16 mm; *p* < 0.001) (Table 2). Moreover, the OBL group showed significantly less preoperative myopia and astigmatism. Although the OBL group had a larger mean CCT, the difference between the two groups did not reach statistical significance (Table 2 and Figure 5F). Overall, UDVA from 1 day to 1 month between the two groups was not statistically different (Table 2).

**Figure 6:**
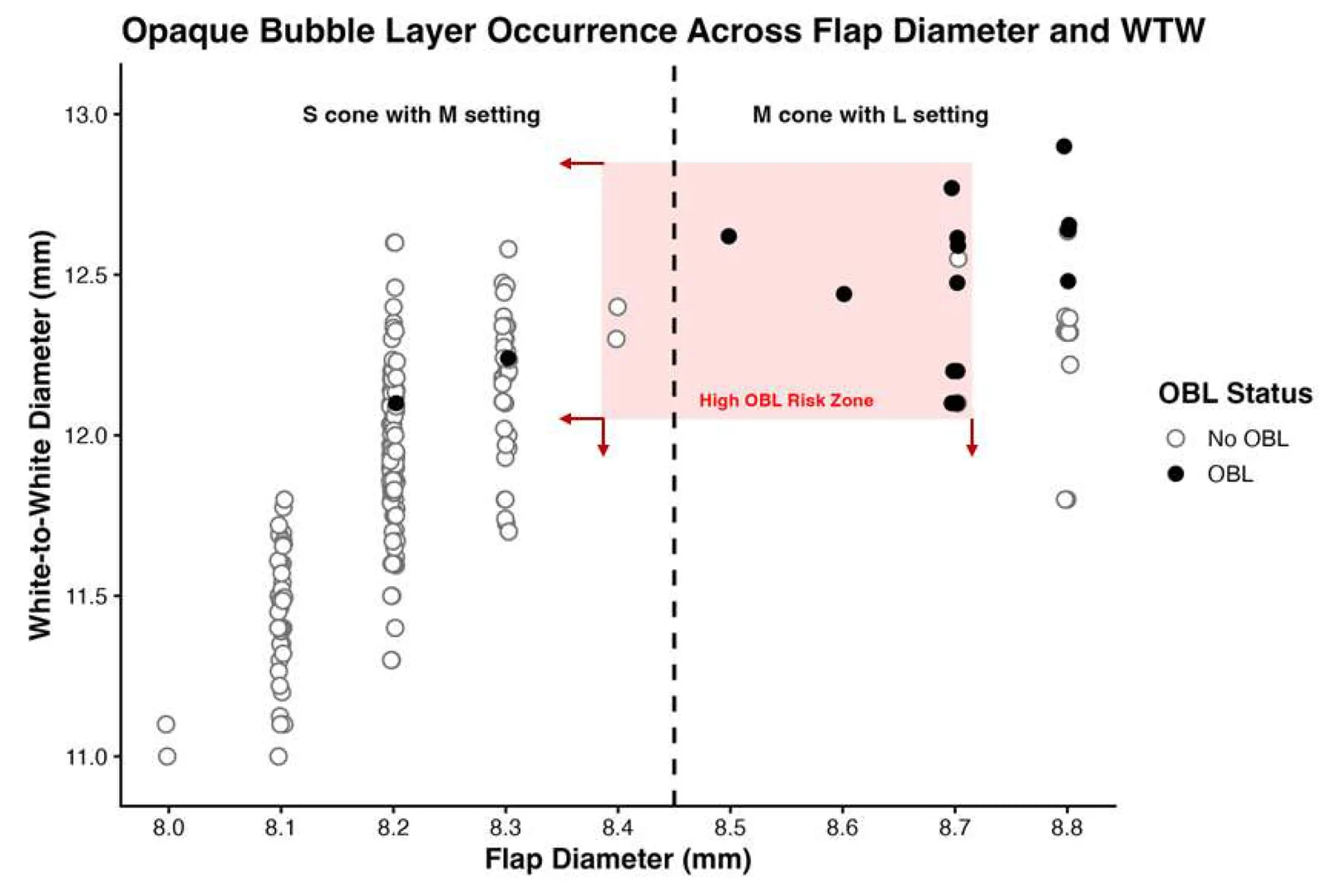
OBL occurrence across flap diameters and WTW. S cones with M settings were utilized for flap diameters between 8.0 and 8.4 mm. M cones with L settings were used for flap diameters of 8.5–8.8 mm. The dashed line divides the two cone settings. The high OBL risk zone depicted by the red rectangle and arrows represents the area with a flap diameter of 8.4–8.7 mm.

**Figure 7:**
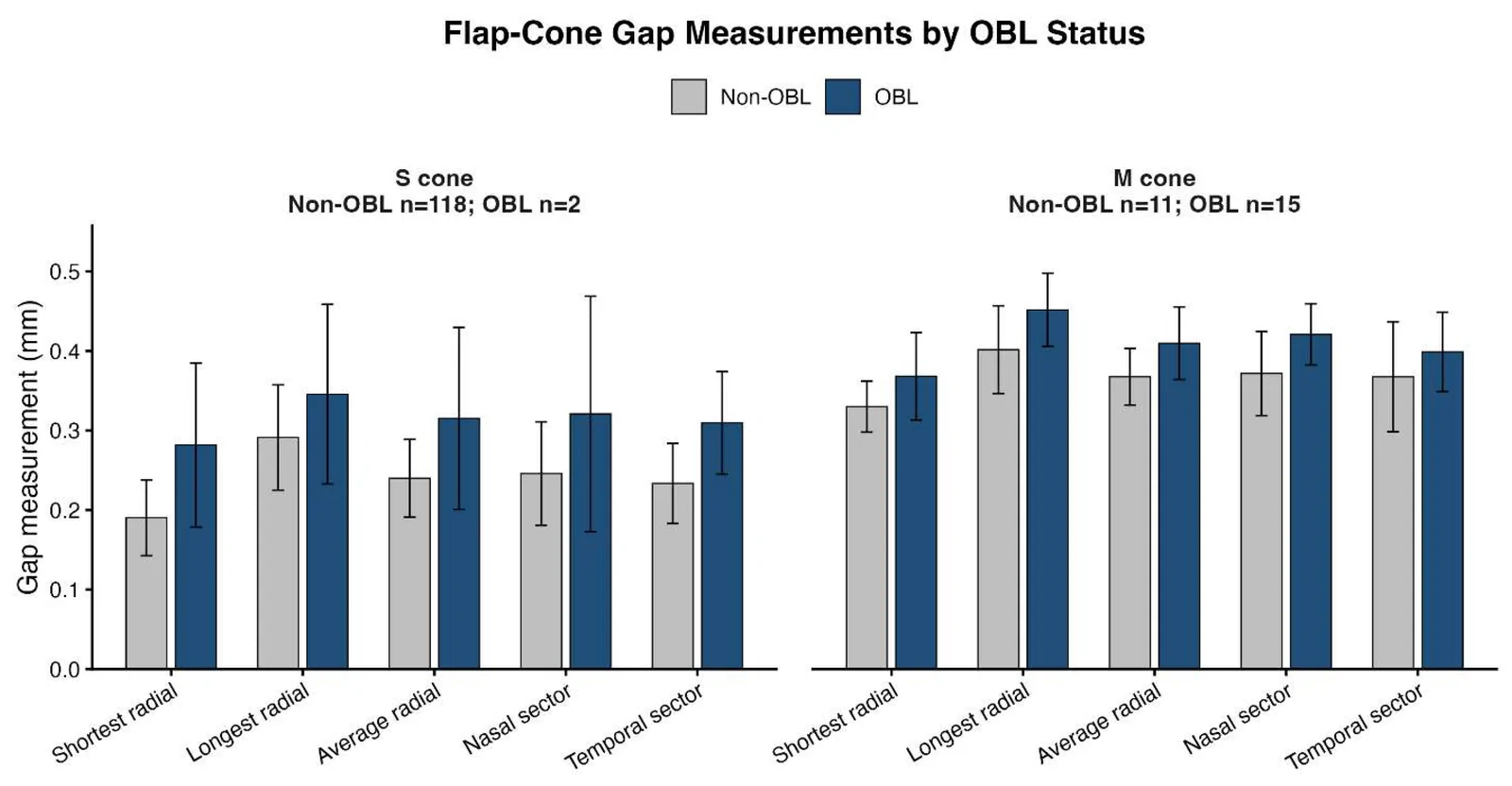
Comparison of mean flap–cone gap measurements by OBL status. Error bars represent standard deviation.

When stratified by cone settings, the majority of OBLs (15/17, 88.2%) occurred in patients with M cones and L settings (Table 3 and Figure 6). Eyes treated with M cones and L settings had significantly larger WTW measurements (12.4 ± 0.3 mm vs. 11.9 ± 0.4 mm; *p* < 0.001) and larger flap diameters (8.7 ± 0.1 mm vs. 8.2 ± 0.1 mm; *p* < 0.001) compared to eyes treated with S cones and M settings. Furthermore, 1-month logMAR SEQ was statistically different between groups (0.10 ± 0.2 vs. −0.05 ± 0.4; *p* = 0.04), while all other refractive outcomes from 1 day to 1 month showed no statistical difference between the two groups. Image analysis also revealed that the average gap between the flap and cone was significantly larger in the OBL group with M cones and L settings (0.41 ± 0.05 mm vs. 0.37 ± 0.04 mm; *p* = 0.007). Notably, the exact nasal gap (0.42 ± 0.04 vs. 0.37 ± 0.05; *p* = 0.02) and nasal sector gap (0.42 ± 0.04 vs. 0.37 ± 0.05; *p* = 0.02) were also significantly larger in the OBL group (Figure 7). In eyes with severe OBLs that could affect pupil centration, excimer laser ablation was delayed until adequate bubble dissipation. Otherwise, eyes with minor OBLs continued with ablation without any additional adjustments. Additional complications were uncommon, including intraoperative suction loss in two cases and postoperative striations in two cases, necessitating a refloat procedure. Both eyes with suction loss were redocked, and the procedure was completed without further complications.

## 4. Discussion

This study evaluates postoperative outcomes of VISUMAX 800 and Allegretto EX500 FS-LASIK. Refractive outcomes were favorable and comparable to VISUMAX 500 FS-LASIK, with 94% of eyes having UDVA 20/20 or greater at 3 months in our cohort (Figure 2A). In comparison, a prior study reported 93% of patients with a postoperative UDVA of 20/20 or better with the VISUMAX 500 femtosecond laser and the EX500 excimer laser [17]. Older studies reported a 75.5–91% rate of UDVA of 20/20 or better with VISUMAX 500, but with different excimer lasers [5,18,19]. This study had 89% and 97% of eyes with an SEQ within ± 0.5 D and ± 1.0 D, respectively, which was also comparable to a previous analysis of VISUMAX 500 FS-LASIK that reported 86.9% of eyes within 0.5 D of attempted correction and 97.3% within 1.0 D of attempted correction [5]. Moreover, the safety index was 1.13 ± 0.17, with no eyes losing any lines of CDVA at 3 months. This matches a prior randomized controlled trial’s findings of VISUMAX 500 FS-LASIK that found a safety index of 1.1 ± 0.2 at 3 months [20]. Together, these results support the efficacy, safety, and predictability of the VISUMAX 800 platform for LASIK flap creation.

Although postoperative outcomes were favorable overall, there were some notable complications such as OBL (17/226; 7.5%) and suction loss (2/226; 0.88%). Topcu et al. also reported favorable refractive and safety outcomes in VISUMAX 800 FS-LASIK that were comparable to SMILE, though surgical timing was not reported [21]. To address this gap, the present study reported favorable mean suction and laser times (15.48 ± 1.87 s and 7.00 ± 0.38 s, respectively). In fact, a prior study specifically analyzing FS-LASIK procedure times using VISUMAX 500 reported mean suction times between 23 and 26 s across the two surgeons [22]. Shorter suction times may improve postoperative outcomes and reduce intraoperative complications, such as decreasing the incidence of intraoperative IOP spikes and suction loss [23]. Interestingly, although we would expect lower rates of suction loss in the VISUMAX 800 given its faster laser and suction times, rates in this study were comparable to those reported with VISUMAX 500 (0.78%; 3/381) [5]. This similarity emphasizes that suction loss risk is multifactorial, driven by factors such as Bell’s reflex, nociceptive reflex, and patient anxiety, among others [24].

OBL incidence in VISUMAX 800 FS-LASIK is difficult to compare directly to VISUMAX 500, especially due to variability in OBL rate reporting, with rates ranging from 5 to 72.6% depending on flap dimensions and surgical technique [6,7,8,22]. In this study, predicted OBL probability increased with larger WTWs (Figure 6), with the OBL group having larger mean WTW measurements. He et al. also noted that larger WTWs were associated with increased OBL formation in VISUMAX 500 FS-LASIK. A possible explanation could be that larger WTWs or corneal diameters create steeper contact with cones due to the suction primarily being centrally located and curvature gradually declining more peripherally, resulting in increased difficulty for the bubble to dissipate out of the edge [25]. Larger WTWs also need larger cones both to prevent suction loss and to provide the larger flap diameters needed for adequate ablation coverage.

In regard to cones, modification techniques were used to increase flap diameter for each cone size, with M cone settings being used in S cones and L cone settings being used in M cones. Prior studies that employed the cone modification technique emphasize the importance of bringing the flap edge closer to the edge of the cone to create more reliably established paths for gas dissipation [26,27]. Conversely, the further the flap diameter is from the cone’s edge, the greater the likelihood of OBL formation. However, the existing literature on cone modification techniques only utilized the S cone with M settings [26,27]. In addition to those parameters, our study utilized M cones with L settings for eyes that needed flap diameters of 8.5 mm or larger.

**Figure 8:**
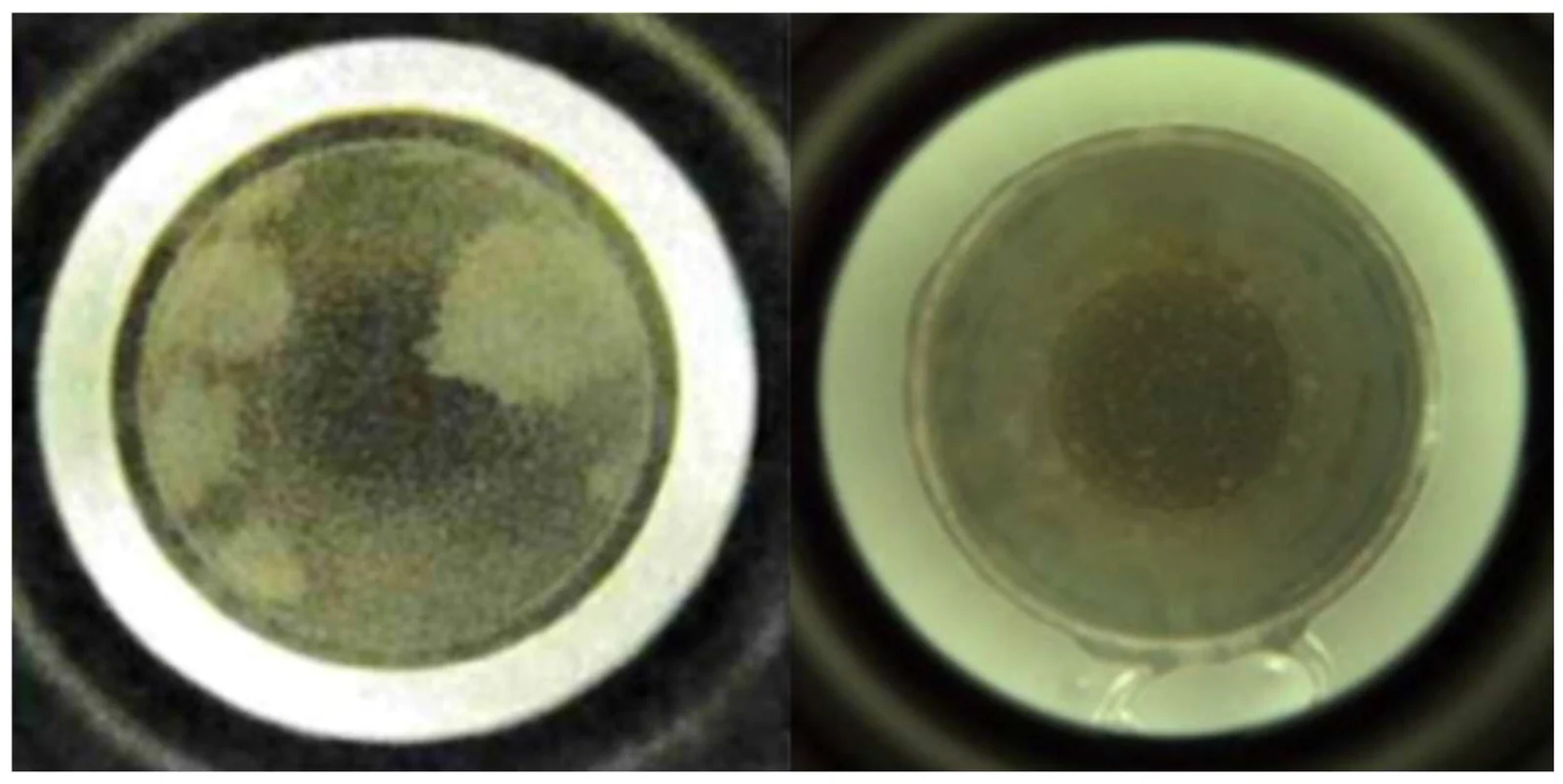
OBL formation with an 8.5 mm flap diameter with M cone and L settings (**left**) and an 8.2 mm flap diameter with S cone and M settings (**right**).

Flap diameters in the range of 8.5–8.6 mm had the highest incidence and severity of OBLs (Figure 8), with flaps closer to the measured cone diameter (9.3 mm) having progressively lower incidence. Interestingly, image analysis revealed that patients who received M cones with L settings and developed OBLs had a significantly larger average gap between flap and cone edge compared to those without OBLs (*p* = 0.007). Larger flaps that approach both the S and M cone diameter appear to have lower OBL incidence compared to flaps of smaller sizes, consistent with the mechanism of reduced flap–cone distance increasing gas dissipation. Although our study found a greater predicted probability of OBLs with increased flap diameter and WTW (Figure 5B,D), this can be attributed to the majority of OBLs occurring in the M cone group, which has significantly larger WTW and flap diameters compared to the S cone group (Table 3; *p* < 0.001).

There is a sparsity in published data regarding the use of flap diameters greater than 8.3 mm with the VISUMAX femtosecond laser platform. Larger flaps are desirable in eyes with greater corneal diameters to ensure adequate ablation coverage. Notably, the existing literature on the VISUMAX 500 reports that larger flap diameters are associated with decreased risk of OBL formation [27]. These reports could be attributed to the majority of studies utilizing S cones and finding a unimodal distribution of OBL formation, with flap diameters below a certain threshold increasing OBL formation, and larger diameters contained within the cone reducing the risk of OBL formation due to proximity to the cone edge. However, our study highlights the possibility of a multimodal distribution of OBLs in relation to the cone/contact glass diameter rather than flap diameter size alone. Additional factors may also play a role in OBL formation, such as docking, flap thickness, and central corneal thickness [6,7,28].

Based on initial surgical experience using the VISUMAX 800 FS-LASIK, the following flap diameter and cone settings may help reduce OBL formation. M cones with L settings should not use flap diameters below 8.8 mm due to high OBL incidence and increased distance from the cone edge. For S cones with M settings, flap diameters below 8.0 will increase risk of OBL formation, with prior studies showing efficacy between 8.0 and 8.2 mm [5,17,27]. Although M cones with M settings and S cones with S settings may be used, OBL rates will likely increase due to a greater distance from the cone edge and a less reliable path for gas dissipation [26]. Conversely, as the distance from the cone edge and flap diameter decreases, one risks having the flap diameter exceed the cone diameter, resulting in an incomplete side cut and flap creation. This is especially critical given that the VISUMAX platform utilizes curved applanation cones, causing the target flap diameter to underestimate the achieved flap diameter, with prior studies reporting up to a 0.47 mm difference between target and achieved flap diameter in VISUMAX 500 [17,29]. Although the concavity of the VISUMAX applanation cone will result in larger flaps, this study keeps the nomenclature consistent with the current literature and utilizes the target pre-laser flap diameter.

Hence, choosing a cone size is a multifactorial decision based on numerous preoperative parameters. Zeiss recommends maximum flap diameters for each cone size, with increasing corneal steepness necessitating smaller flap diameters across all cone sizes [30]. In our experience, flap diameters increase stepwise based on WTW corneal diameter as well, but WTWs between 12.2 and 12.5 mm have no optimal flap diameter due to the OBL risk (Table 4).

OBL formation can disturb complete flap creation in addition to increasing the risk of the eye tracker being interrupted due to difficulty detecting the pupillary center or visual axis [7,26]. This may result in decentered ablation, tracking errors during the procedure, or deeper migration of bubbles leading to vertical gas breakthrough and subsequent complications such as subepithelial cell growth [31]. As a result, surgeons may choose to delay procedure completion to allow for adequate OBL dissipation for certain cases, interrupting surgical flow and increasing patient anxiety. Although reducing OBL formation is critical to improving surgical flow, OBLs showed little impact on visual outcomes from 1 day to 1 month in our study (Table 2). This coincides with the existing literature that finds that most OBLs do not affect long-term visual outcomes [6,7].

Due to the cone modification technique, no direct conclusion can be drawn that links larger WTW or flap diameters to increased OBL risk by themselves. Instead, these variables must be factored in conjunction with other surgical parameters such as cone size to better assess OBL risk, due to the interrelated nature of these variables in preoperative planning. Our results suggest that the relationship between flap diameters and OBLs might be multimodal, with incidence peaks dependent on the cone/contact glass utilized. In the present study, flap diameters between 8.4 and 8.7 mm had an increased risk of severe OBL formation, with no available cone or cone modification technique being able to reduce OBL rates in this range. With modern-day excimer lasers usually having a total treatment zone of 8.5 to 9 mm in diameter, the target flap diameter should factor in the expected ablation zone [32]. Given that the actual VISUMAX platform flap diameters are larger than target diameters due to the concavity of the cones, the target flap diameter range of 8.5 to 8.7 mm can give adequate coverage for most excimer lasers. With the current S and M cones measuring 8.7 and 9.3 mm, respectively, expanding the available cone options to include intermediate sizes such as 9.0 mm may help address this dilemma. Further clarification is needed to better identify methods of overcoming this limitation to allow for flap diameters in this transitional range to be used for appropriate patients.

The single-center retrospective nature of the study limits the sample size and generalizability of the results. Additionally, the shorter follow-up period, compounded with the number of eyes lost to follow-up, further introduces potential for bias. Another limitation was the subjective nature of the manual cone measurements and digital image analysis of flap–cone gaps, which introduces measurement variability. Because each image was reviewed by a single researcher, inter-rater reliability was not obtained. Although OBL formation was more frequent at flap diameters of 8.5 and 8.6 mm, each of these diameters was represented by a single eye in our study, which limits the significance of this finding. These diameters fell outside the recommended range for the corresponding cones because of high OBL incidence and were therefore used infrequently. The sample size of 17 OBL eyes limited meaningful multivariable analysis to adjust for other possible contributors to OBL formation such as docking, flap thickness, or central corneal thickness. Therefore, these findings should be regarded as preliminary observations that require further confirmation with larger cohorts.

## 5. Conclusions

In our initial experience with the VISUMAX 800 FS-LASIK, the device demonstrated favorable safety and efficacy, comparable to the VISUMAX 500 but with improved surgical times. OBLs were the most common intraoperative finding, particularly in cases that had M cones with L settings, with larger flap diameters and WTWs disproportionately affected. Our findings support the use of VISUMAX 800 in FS-LASIK and hypothesize a multimodal distribution of OBL risk based on the distance between flap and cone edge. Although VISUMAX is an excellent platform with favorable refractive outcomes in FS-LASIK, we found an inability to create flaps between 8.4 and 8.7 mm in diameter without risking severe OBL formation. Nevertheless, further research is needed to confirm these preliminary findings, elucidate the exact mechanisms underlying these findings, assess long-term outcomes and complications, and identify strategies to overcome this limitation.

## Figures and Tables

**Table 1:** Baseline characteristics of VISUMAX 800 assisted LASIK patients. Values shown as mean ± standard deviation.

Characteristic	Value
Total patients/eyes	117 patients/226 eyes
OD/OS, *n* (%)	115 (51%)/111 (49%)
Age (y)	33.99 ± 8.50 (20.00–58.00)
Male/female, *n* (%)	53 (45.3%)/64 (54.7%)
OBL yes/no, *n* (%)	17 (7.5%)/209 (92.5%)
Flap diameter (mm)	8.27 ± 0.19 (8.00–8.80)
Flap thickness (µm)	100.18 ± 1.23 (100.00–110.00)
WTW average (mm)	11.93 ± 0.38 (11.00–12.90)
Preoperative sphere	−2.81 ± 1.81 (−7.75–0.75)
Preoperative cylinder	−1.08 ± 1.02 (−5.00–0.00)
Preoperative SEQ	−3.35 ± 1.74 (−7.88–0.00)
K1 (D)	43.22 ± 1.33 (39.60–46.70)
K2 (D)	44.45 ± 1.41 (41.20 to 48.30)
Pachymetry (µm)	555.95 ± 26.79 (506.00–631.00)
Suction duration (s)	15.48 ± 1.87 (9.00–27.00)
Laser duration (s)	7.00 ± 0.38 (3.00–8.00)

WTW = white to white; K1 = flat meridian; K2 = steep meridian.

**Table 2:** Preoperative characteristics of patients with and without OBL formation.

Variable	Non-OBL Group	OBL Group	GEE *p*-Value
Total eyes	209	17	N/A
Age (y)	33.80 ± 8.59 [20.00 to 58.00]	36.35 ± 6.98 [26.00 to 50.00]	0.343
Laser duration (s)	6.99 ± 0.38 [3.00 to 8.00]	7.12 ± 0.33 [7.00 to 8.00]	0.263
Preoperative SEQ (D)	−3.39 ± 1.69 [−7.88 to 0.00]	−2.78 ± 2.19 [−6.50 to −0.12]	0.003
Preoperative cylinder (D)	−1.10 ± 1.04 [−5.00 to 0.00]	−0.76 ± 0.68 [−2.50 to 0.00]	0.032
Preoperative K1 (D)	43.25 ± 1.34 [39.60 to 46.70]	42.82 ± 1.12 [41.20 to 45.20]	0.251
Preoperative K2 (D)	44.50 ± 1.42 [41.20 to 48.30]	43.81 ± 1.13 [42.20 to 46.60]	0.064
Preoperative pachymetry (µm)	555.47 ± 26.78 [506.00 to 631.00]	561.82 ± 26.98 [529.00 to 618.00]	0.463
WTW (mm)	11.89 ± 0.36 [11.00 to 12.63]	12.42 ± 0.26 [12.10 to 12.90]	<0.001
Flap diameter (mm)	8.23 ± 0.16 [8.00 to 8.80]	8.65 ± 0.17 [8.20 to 8.80]	<0.001
Flap thickness (µm)	100.19 ± 1.28 [100.00 to 110.00]	100.00 ± 0.00 [100.00 to 100.00]	0.079
1-day UDVA logMAR	0.02 ± 0.14 [−0.12 to 0.88]	0.01 ± 0.04 [−0.10 to 0.11]	0.514
1-week UDVA logMAR	0.00 ± 0.14 [−0.12 to 0.70]	−0.00 ± 0.08 [−0.12 to 0.10]	0.878
1-month UDVA logMAR	−0.03 ± 0.11 [−0.19 to 0.54]	−0.01 ± 0.05 [−0.10 to 0.06]	0.308
1-month CDVA logMAR	−0.05 ± 0.06 [−0.19 to 0.13]	−0.04 ± 0.05 [−0.12 to 0.00]	0.243
1-month safety index	1.14 ± 0.17 [0.87 to 1.75]	1.12 ± 0.14 [1.00 to 1.33]	0.507
1-month efficacy index	1.10 ± 0.23 [0.29 to 1.88]	1.05 ± 0.13 [0.87 to 1.25]	0.220

Values are shown as mean ± standard deviation [range]. *p*-values were derived from generalized estimating equations. *p* < 0.05 was considered statistically significant.

**Table 3:** Comparison between S cone/M settings and M cone/L settings groups.

Variable	S cone/M Settings (OBL = 2/198 Eyes, 2 Patients)	M Cone/L Settings (OBL = 15/28 Eyes, 9 Patients)	GEE *p*-Value
Age (y)	34.02 ± 8.59 [21.00 to 58.00]	33.79 ± 7.93 [20.00 to 45.00]	0.745
Laser duration (s)	6.99 ± 0.39 [3.00 to 8.00]	7.07 ± 0.26 [7.00 to 8.00]	0.276
Preoperative SEQ (D)	−3.44 ± 1.75 [−7.88 to 0.00]	−2.65 ± 1.52 [−5.75 to −0.12]	0.087
Preoperative cylinder (D)	−1.06 ± 0.95 [−4.50 to 0.00]	−1.18 ± 1.40 [−5.00 to 0.00]	0.733
Preoperative K1 (D)	43.30 ± 1.31 [39.60 to 46.70]	42.62 ± 1.29 [41.00 to 45.70]	0.057
Preoperative K2 (D)	44.52 ± 1.38 [41.20 to 48.30]	43.92 ± 1.50 [41.50 to 47.30]	0.141
Preoperative pachymetry (µm)	555.63 ± 26.53 [506.00 to 623.00]	558.21 ± 28.97 [526.00 to 631.00]	0.747
WTW (mm)	11.87 ± 0.35 [11.00 to 12.60]	12.37 ± 0.27 [11.80 to 12.90]	<0.001
Flap diameter (mm)	8.20 ± 0.07 [8.00 to 8.40]	8.74 ± 0.07 [8.50 to 8.80]	<0.001
Flap thickness (µm)	100.15 ± 1.12 [100.00 to 110.00]	100.36 ± 1.89 [100.00 to 110.00]	0.561
1-day UDVA logMAR	0.02 ± 0.14 [−0.12 to 0.88]	0.00 ± 0.06 [−0.12 to 0.15]	0.292
1-week UDVA logMAR	0.00 ± 0.14 [−0.12 to 0.70]	−0.02 ± 0.09 [−0.12 to 0.11]	0.448
1-month SEQ	−0.05 ± 0.39 [−1.62 to 0.75]	0.10 ± 0.24 [−0.25 to 0.62]	0.040
1-month UDVA logMAR	−0.02 ± 0.11 [−0.19 to 0.54]	−0.02 ± 0.07 [−0.12 to 0.11]	0.995
1-month CDVA logMAR	−0.05 ± 0.06 [−0.19 to 0.13]	−0.05 ± 0.06 [−0.12 to 0.00]	0.980
1-month safety index	1.14 ± 0.18 [0.87 to 1.75]	1.13 ± 0.15 [1.00 to 1.44]	0.843
1-month efficacy index	1.10 ± 0.23 [0.29 to 1.88]	1.08 ± 0.16 [0.85 to 1.44]	0.608

All values are reported as mean ± standard deviation [range]. *p*-values were derived from generalized estimating equations. *p* < 0.05 was considered significant.

**Table 4:** Preliminary experience of flap diameter selection based on WTW corneal diameters.

WTW (mm)	Flap Diameter (mm)
>12.5	>8.8
12.2–12.5	No reliable flap diameter
11.7–12.2	8.2–8.3
<11.7	<8.2

## Data Availability

The original contributions presented in this study are included in the article. Further inquiries can be directed to the corresponding author (M.M.).
